# Peptide P5 (residues 628–683), comprising the entire membrane proximal region of HIV-1 gp41 and its calcium-binding site, is a potent inhibitor of HIV-1 infection

**DOI:** 10.1186/1742-4690-5-93

**Published:** 2008-10-16

**Authors:** Huifeng Yu, Daniela Tudor, Annette Alfsen, Beatrice Labrosse, François Clavel, Morgane Bomsel

**Affiliations:** 1 Entrée Muqueuse du VIH et Immunité Muqueuse, (Mucosal Entry of HIV-1 and Mucosal Immunity), Departement de Biologie Cellulaire, (Cell Biology Department), Institut Cochin, Université Paris Descartes, CNRS (UMR 8104), 22 rue Mechain, 75014 Paris, France; 2 Inserm, U567, Paris, France; 3 Inserm U552 Hopital Bichat-Claude Bernard, 46, rue Henri Huchard, 75018 Paris, France; 4 Université Paris Diderot, Paris, France

## Abstract

The membrane proximal region (MPR) of the transmembrane subunit, gp41, of the HIV envelope glycoprotein plays a critical role in HIV-1 infection of CD4^+^ target cells and CD4-independent mucosal entry. It contains continuous epitopes recognized by neutralizing IgG antibodies 2F5, 4E10 and Z13, and is therefore considered to be a promising target for vaccine design. Moreover, some MPR-derived peptides, such as T20 (enfuvirtide), are in clinical use as HIV-1 inhibitors. We have shown that an extended MPR peptide, P5, harbouring the lectin-like domain of gp41 and a calcium-binding site, is implicated in the interaction of HIV with its mucosal receptor. We now investigate the potential antiviral activities of P5 and other such long MPR-derived peptides. Structural studies of gp41 MPR-derived peptides using circular dichroism showed that the peptides P5 (a.a.628–683), P1 (a.a.648–683), P5L (a.a.613–683) and P7 (a.a.613–746) displayed a well-defined α-helical structure. Peptides P5 inhibited HIV-1 envelope mediated cell-cell fusion and infection of peripheral blood mononuclear cells by both X4- and R5-tropic HIV-1 strains, whereas peptides P5 mutated in the calcium binding site or P1 lacked antiviral activity, when P5L blocked cell fusion in contrast to P7. Strikingly, P5 inhibited CD4-dependent infection by T20-resistant R5-tropic HIV-1 variants. Cell-cell fusion studies indicated that the anti-HIV-1 activity of P5, unlike T20, could not be abrogated in the presence of the N-terminal leucine zipper domain (LZ). These results suggested that P5 could serve as a potent fusion inhibitor.

## Introduction

In the vast majority of cases, HIV-1 transmission occurs at mucosal sites. The initial target cells for HIV-1 at mucosal sites include epithelial cells (CD4-negative) in simple monostratified mucosa (rectum, gastrointestinal tract, endo-cervix) and dendritic cells in pluristratified mucosa (vagina, exo-cervix, foreskin). Entry of HIV-1 into both types of cells is mediated by the cooperative interaction between both HIV-1 envelope subunits, gp120 and gp41, and galactosyl ceramide (GalCer) [1-3], thereby inducing HIV endocytosis in target cells and subsequent transcytosis or transfer to susceptible CD4^+^ T cells [4]. We have previously demonstrated that the peptide P1 (a.a. 649–683) derived from the membrane proximal region (MPR) of gp41 acts as a galactose-specific lectin in binding to GalCer, the HIV-1 mucosal receptor expressed on both epithelial and dendritic cells [2,4,5]. In this case, HIV-1 neither fuses with nor infects target cells.

In contrast, CD4^+^ T cells are infected by HIV-1, leading to HIV spread. Infection is mediated by the HIV-1 envelope glycoproteins gp120/gp41, which trigger fusion between viral and cellular membranes, resulting in productive infection. Viral replication then causes rapid CD4^+^ T cell depletion, essentially at mucosal sites. Upon binding to CD4 and the co-receptor CCR5/CXCR4, gp120 undergoes serial conformational changes that allow the insertion of the gp41 fusion peptide into the target cell membrane and formation of the pre-hairpin structure. Subsequent formation of a hairpin structure (six-helix-bundle) promotes fusion between viral and cellular membranes [6,7]. The hydrophobic region of the MPR plays an important role in this conformational change [8,9]. The membrane fusion step can be inhibited by peptides mimicking the sequence of N-terminal (NHR) or C-terminal (CHR) heptad repeats, which block the association of the NHR and CHR regions, thus preventing hairpin formation [10].

In sum, the highly conserved MPR of gp41, which contains continuous epitopes recognized by broadly neutralizing antibodies 2F5 [11], 4E10 [12] and Z13 [13], appears to be essential for both CD4-dependent target cell infection and CD4-independent mucosal entry of HIV-1. The MPR, along with the C-terminal cytoplasmic tail, is known to be determinant for envelope glycoprotein (Env) incorporation into virions and virus infectivity [9,14].

In general, peptides from the CHR region (C-peptides) display higher inhibitory activity than peptides from the NHR region (N-peptides) [15]. The first approved fusion inhibitor drug, Enfuvirtide (T20, a.a.640–673), displays an IC50 value in the nM range against some laboratory-adapted HIV-1 isolates *in vitro*, and excellent efficacy in clinical trials [16-18]. However, it leads *in vivo *to the generation of viral escape mutants, restricting its potential use for therapeutic purposes [19].

Peptide P1 is the minimal region of the MPR allowing interaction with GalCer. It contains three subdomains essential for its lectin activity, namely, the CHR which is rich in glutamic acid and highly negatively charged, the central hexapeptide ELDKWA epitope recognized by the potent and broadly neutralizing 2F5 IgG [11], and a hydrophobic tryptophan-rich region recognized by the other gp41-specific broadly neutralizing IgG, 4E10 and Z13 [12,13]. Our recent biophysical studies [20] of peptides P1 and P5 (a.a.628–683), revealing an extended structure comprising not only the MPR peptide, but also the gp41 calcium-binding site (a.a.628–648) in its N-terminal portion [21], suggested that the affinity of gp41 for GalCer is dependent on the conformation of its lectin-binding site, which depends upon its environment. In particular, in the presence of calcium P5 undergoes a secondary structural change that decreases its affinity for GalCer.

In an effort to develop an effective microbicide, vaginal application of a peptide (C52L) similar to P5 [22], in combination with the CCR5 ligand CMPD167 or the entry inhibitor BMS378806, afforded protection against infection in Rhesus macaques upon vaginal challenge with SHIV.

As P1 and P5 comprise a larger portion of the MPR and were crucial for HIV entry in mucosa, we have evaluated in the present study their antiviral activity against HIV-1-mediated fusion and infection in comparison with T20, which comprises a more restricted region of the MPR.

**Figure 1:**
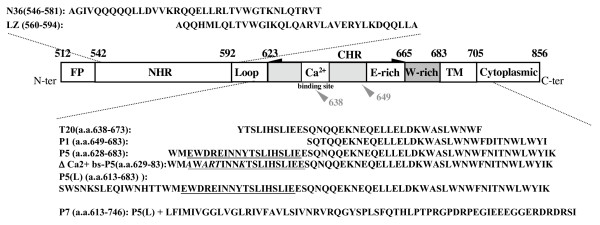
**Schematic representation of the HIV-1 gp41 domains**. Gp41 comprises cytoplasmic, transmembrane (TM) and extracellular domain. Important functional extracellular-domains of gp41 include the fusion peptide (FP), N-terminal and C-terminal heptad repeats (NHR or HR1 and CHR or HR2, respectively). Peptides used in the present study are as follows: P5 (a.a.628–683), Δ Ca2+ bs-P5 (a.a. 628–683) corresponding P5 mutated (bold characters) in the calcium-binding site, P1 (a.a. 648–683), P5L (a.a. 613–683), P7 (a.a.613–746), N36 (a.a. 546–581) LZ (a.a. 560–594). T-20 (a.a. 638–673) is a well-described HIV-1 fusion inhibitor (Enfurvirtide). The residues are numbered according to their position in gp160 of HIV-1_HXB2_. The N-ter of T20 (aa 638) located within the calcium-binding site and that of P1 (a.a. 649) are indicated (grey arrow head). The calcium-binding site in P5 and P5 (L) are underlined.

In the present study, the anti-HIV-1 activity and the structure of the different MPR-derived peptides, including P1 (a.a.648–683), P5 (a.a.628–683), P5L (a.a.613–683) and P7 (a.a.613–746), were investigated. The schematic representation and the sequence of these peptides are presented in Figure 1. The P5L peptide comprises P5 and a caveolin-binding domain -1, the target of neutralizing antibodies [23]. The P7 peptide contains the transmembrane region (TM) and part of the C-terminal tail, the latter including sequences reported to be the target of neutralizing antibodies [24,25].

## Methods

### Materials

Polymerase and deoxynucleoside triphosphates (dNTPs) for PCR amplification and isopropyl-beta-D-thiogalactopyranoside (IPTG) were purchased from Invitrogen. IgG sepharose was from Amersham Bioscience (Paris, France). Bacterial cells (*E. coli *Rosetta™) were from Novagen. The eukaryotic expression vector pGEV, designed for improving expression [26], was provided by Drs. F. Toma and P. Curmi (Evry-Val d'Essonne University, Evry, France). Materials used to purify PCR products were from QIAGEN (Paris, France). Ligase and restriction enzymes *(Nco*I and *Not*I) were from New England Biolabs, Inc (MA, USA). All other chemicals were of the highest purity commercially available (Sigma).

Chemically synthesized peptides P5 (628–683), P5 mutated in the calcium binding site (Δ Ca2+ bs-P5, 629–683), P1 (649–683) and LZ (560–594) were from NeoMPS (France) and Eurogentec (Belgium) with a purity >90%. Peptides T20 (# 9845) and N36 (546–581) (# 9773) were obtained from the AIDS Research and Reference Reagent Program, NIAID.

### Construction, expression and purification of recombinant peptides

The coding sequence of all peptides was PCR amplified by use of a pair of synthetic oligonucleotides chosen in reference to the cDNA sequence of HIV_HXB2_. The PCR products were subsequently digested with *Nco*I/*Not*I and cloned into the pGEV vector, previously digested at the same sites. The sequences of recombinant constructs were confirmed by restriction enzyme analysis and DNA sequencing using the dideoxy chain termination method.

The recombinant plasmids pGEV-P1, P5, P5L and P7 bearing the transmembrane region (TM) were transformed into *E. coli *Rosetta™ (Novagen) competent cells by the heat shock procedure. One liter of LB medium (containing 100 μg/mL ampicillin) was inoculated with 5 mL of a fresh overnight culture and incubated at 37°C. The cultures were induced by the addition of 1 mM isopropyl-β-D-thiogalactopyranoside (IPTG) at an OD600 of 0.5–0.7, and further incubated for 3 h or overnight, under the same conditions. The cells were then harvested by centrifugation at 6,000 rpm for 20 min at 4°C. The pelleted cells were suspended in 100 mL of TST Buffer (50 mM Tris, 150 mM NaCl, 0.05% Tween-20; pH = 7.6), and sonicated. Cell debris was eliminated by centrifugation at 10,000 × *g *for 20 min. The soluble fraction was incubated for 2 h at 4°C in 5 ml of IgG-Sepharose that had been equilibrated in Buffer TST. The beads were washed once with 10 column volumes of TST Buffer, and subsequently 3 times with 10 column volumes of washing buffer (5 mM NH_4_Ac, pH = 5.0). Peptides were then eluted with 0.5 M CH_3_COOH, pH = 3.3 and immediately dialyzed against water or PBS, overnight.

### Reverse Phase Chromatography

Reverse phase high performance liquid chromatography (RP HPLC) was performed using a C18 column. Peptides were eluted with buffer A (0.1% trifluoroacetic acid, 5% acetonitrile) for 15 min followed by a linear gradient from 0 to 100% of buffer B (0.1% trifluoroacetic acid, 80% acetonitrile) over 5 min, while monitoring by absorbance at 280 or 218 nm. The peaks corresponding to peptides P1 or P5 were further analyzed using MALDI-TOF Mass Spectrometry [27].

### Far-UV circular dichroism measurements

Far-UV circular dichroism (CD) was performed using 10 μM of each peptide solution in PBS. Each spectrum was recorded from 260 nm to 190 nm at 25°C by using a Jasco CD spectrophotometer (Jasco, Japan) with a cell path length of 0.05 cm. Spectra were collected as an average of three scans, with a scan speed of 20 nm/min and a response time of 2 sec. The control (solvent) CD spectra were subtracted to eliminate background effects. Data were reported [28] in millidegrees and converted to molar ellipticity [θ] in units as follows: degree cm^2 ^dmol^-1^. The percentage of α-helical content was estimated by assuming that a [θ]222 value of -33000 deg cm^2 ^dmol^-1^corresponds to 100% α-helix.

### Construction of viruses carrying primary envelope glycoproteins

We constructed viruses bearing the envelope glycoproteins derived from virus populations present before and during T20 treatment of a patient who received T20 as part of a salvage therapy, as we reported earlier. Hence, a portion of the envelope gene encoding gp120 and the ectodomain of gp41 (nt 6480 to 8263), previously subcloned into the pCR2.1TOPO vector, was amplified with primers that allowed the introduction of two restriction enzyme sites (AgeI at position 6346 and NheI at position 8287) and digested. Each chimeric proviral clone was obtained by replacing the AgeI-NheI fragment of a modified pNL4-3 vector (nt 6346 to 8287 in pNL4-3) by a AgeI-NheI PCR fragment amplified from the envelope subclone. Sequences of oligonucleotides used for PCR and site-directed mutagenesis can be provided upon request. The pre-T20 virus expressing envelope glycoproteins derived from the pretherapy virus population did not carry the well characterized T20-resistance mutations, which are for the most part located between residues 36 to 45 of the NHR region of gp41 [Sista, 2002 #47] [29]. The envelope proteins of the two other viruses, T20-1 and T20-2, as well as the virus populations from which they were derived, expressed the V38A resistance mutation [30].

### Cells and virus stocks

Hela-CD4-LTR-LacZ cells (P4.2, kindly provided by Dr. M. Alizon, Institut Cochin, Paris, France) stably express human CD4 and CXCR4, as well as the β-galactosidase-encoding gene lacZ under the transcriptional control of the HIV-1 long terminal repeat (LTR), which is activated by HIV-1 Tat [31]. Hela-env-Lai cells stably express HIV-Lai *env *genes from X4 virus and Tat in the cytosol. All cell lines were cultured in Dulbecco's modified Eagle medium (DMEM, GIBCO) containing physiological concentration of calcium (1.8 mM) supplemented with 10% heat-inactivated fetal calf serum (FCS), 100 U/ml penicillin, 100 mg/ml streptomycin and 2 mM glutamine. The human cell line U373MG-CD4-CCR5-LTR-lacZ, stably expressing the human receptors CD4 and CCR5 and bearing the lacZ indicator cassette [32], was propagated in DMEM supplemented with 60 μg/ml penicillin, 100 μg/ml streptomycin, and 10% heat-inactivated fetal calf serum. Peripheral blood mononuclear cells (PBMC) were isolated from heparinized human blood by Ficoll (Ficoll-Paque PLUS, Amersham Bioscience) gradient centrifugation and resuspended in fetal calf serum supplemented with 10% DMSO for storage in liquid nitrogen PBMC were activated by incubation in RPMI 1640 medium containing 5 μg/ml phytohemagglutinin-L (PHA-L, Sigma), 100 U/ml penicillin, 100 mg/ml streptomycin, 2 mM glutamine and 10% FCS. After three days of incubation with PHA, cells were washed and used for infection. All cells were cultured in 5% CO_2 _incubators at 37°C. Stocks of HIV-1 strains JRCSF (R5 virus) and Lai (X4 virus) were produced by transfection of 293 T cells by the calcium phosphate method and stored at -80°C [4]. Chimeric HIV-1 particles expressing the gp120 and gp41 ectodomain of patient origin and the cytoplasmic domain of gp41 from pNL4.3 were produced as previously described [30]. Culture supernatants were collected 40 h after transfection. HIV-1 p24 antigen production was quantified for each viral stock by enzyme-linked immunosorbent assay (Kit p24 Innogen, Innogenetics/Ingen).

### HIV-1 env-mediated cell-to-cell fusion assay

Hela-env-Lai cells were seeded in 48-well plates (10^5 ^cells per well) in the presence of peptides (P1, P5, P5L, P7, T20) at concentrations ranging from 50 to 1000 nM. Fifteen minutes later, target cells (HeLa P4.2) were added to the wells (10^5 ^per well) and co-cultured for 6 h at 37°C. Then, cell-cell fusion was monitored by beta-galactosidase assay [33]. Briefly, cells were lysed in 80 μl of lysis buffer (5% NP40 in PBS without CaCl_2 _and MgCl_2_) for 10 min and then centrifuged at 10,000 g for 5 min at 4°C. Equal volumes (50 μl) of lysate and reaction buffer (120 mM Na_2_HPO_4_, 80 mM NaH_2_PO_4_, 2 mM MgSO_4_, 20 mM KCl, 10 mM β-mercaptoethanol, 16 mM CPRG (chlorophenol red-β-D-galactopyranoside, Roche, France) were combined and incubated for 60 min at room temperature in the dark. The absorbance at 590 nm of each sample was measured.

### Neutralization of HIV-1 infection of PBMC

Neutralization of HIV-1 infectivity for PBMC was performed as previously described [34] by analysing intracellular staining of p24 Ag after a single round of infection. Briefly, equal volumes (25 μl) of HIV-1_Lai _or HIV-1_JRCSF _virus (the amount of virus added per well corresponds to 50 ng of p24) and peptides T20, P1 or P5 at the indicated concentration were incubated together for 1 h at 37°C. PHA-stimulated PBMC (25 μl, 2 × 10^5 ^cells/well) were added to the mixture. After overnight incubation, 100 μl of culture medium containing 20 U/ml of IL-2 were added and cells were cultured for an additional 24 h at 37°C. For intracellular p24 Ag staining, cells were fixed with 4% paraformaldehyde for 20 min, permeabilized with 0.05% saponin and stained with a fluorescent anti-p24 mAb (PE-anti-p24, KC57 clone, Beckman Coulter, Hialeah, FL) as a 1/160 dilution in 1% BSA, 0.05% saponin in PBS (pH = 7.4) for 15–20 min at 4°C. The percentage of p24 positive cells was monitored by flow cytometry (FACScalibur with the XL software) by gating 20,000 events on the living cell population identified by forward- and side-scatter parameters.

### Inhibition of T20-resistant HIV-1 strains by P5 and T20

Two T20-sensitive (pre- Enfurvitide (ENF) and JRCSF) and two T20-resistant strains (ENF-1 and ENF-2) were assayed for their susceptibility to P5 and T20. These assays were performed as previously described [30]. Two days before infection, U373MG-CD4-CCR5-LTR-lacZ cells were seeded into 96-well plates at a density of 2,000 cells/well. Infection with 10 ng of p24/well of fresh viral suspension was carried out in triplicate, in the absence or the presence of increasing concentrations of T20 (from 1 to 3,3750 nM) (T20; American Peptide Company, Inc), and of P5 (from 1 to 2,000 nM). Forty hours after infection, cells were lysed using 100 μl/well of lysis buffer (5 mM MgCl2 and 0.1% NP-40 in 1× phosphate-buffered saline), prior to addition of 100 μl/well of chromogenic substrate (6 mM chlorophenol red-β-D-galactopyranoside; CPRG, Roche) in lysis buffer. The concentrations of T20 inhibiting virus infectivity by 50% (IC_50 _values) were calculated by using the median-effect equation [35].

### Binding of Peptides to CD4^+^-target cells

Binding of peptides was evaluated using either the lymphocytic cell line CD4^+^- CEM-NK^r ^(NK-resistant) cells (NIH AIDS Reagent Programm, USA)or PHA-activated CD4^+ ^T cells purified from peripheral blood of 5 healthy donors using human CD4^+^ T cells enrichment kit (StemCell Technologies Inc., France). 5 × 10^5 ^cells were incubated with indicated concentrations of peptide for 1 h at room temperature in a total volume of 50 microliter/μl of RPMI 10%FCS. After 2 wahses in cold medium, bound peptide was detected with the human IgG 2F5 (10 microg/μg), or irrelevant human IgG as control, for 1 h at 37°C followed anti-human IgG-FITC mAb (1:1000, Jackson ImmunoResearch, France). Binding of peptides to CD4^+^ cells was evaluated by flow cytometry (FACSCalibur, Becton Dickinson) on 10^4 ^events, gated by forward and side scatter and analyzed for FL1 (FITC channel) using the Cytomix RXP software. Data are presented as peptide-bound cells % total gated cells to which nonspecific background evaluated with the control IgG was deduced (background value less than 10% of the total counts).

## Results and discussion

In previous studies, we have investigated the structure-function relationship of long synthetic peptides derived from the extracellular domain of gp41. Such long peptides are highly hydrophobic and not easy to produce by chemical synthesis. Furthermore, the role of the transmembrane region of gp41 on the MPR structure and function cannot be analyzed. We therefore chose to express the gp41-derived peptides P1, P5, P5L and P7 in fusion with a cleavable carrier by using a prokaryote expression vector (pGEV) (see Additional file 1).

### Secondary structure analysis of gp41-derived peptides by far UV CD spectra

**Figure 2:**
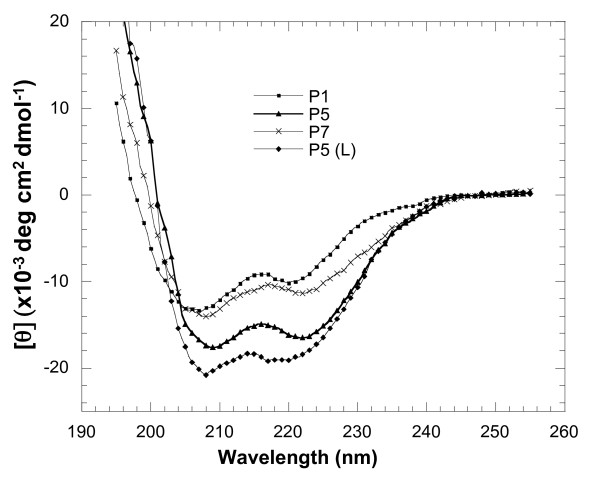
**CD spectra of peptides P1, P5, P5L, P7**. Far-UV CD spectra were all recorded at room temperature, with a path-length of 0.05 cm. Peptide concentration was 10 microM in PBS, pH 7.2. For each spectrum, the medium base line has been subtracted and mean residue ellipticity (degree^.^centimeter^2.^decimole^-1^) versus wavelength is shown. Every measurement in this figure was performed three times and one representative set of spectra is shown.

CD spectroscopy is a practical and efficient method for secondary structure characterization of a protein or peptide. CD spectra of the synthetic peptides P1, P5, and recombinant peptides P5L and P7, at a concentration of 10 μM and in a pH 7.2 buffer, displayed a positive peak after 195 nm and two negative maxima at 208 nm-222 nm, characteristic of α-helices (Figure 2). Quantification of the α-helical content of these peptides indicated that the peptides P5 and P5L exhibited higher helical content (49.9% and 56.6% respectively) than peptides P1 and P7 (30.3% and 34.5%, respectively).

In order to compare the effect of the addition of NHR peptides to P5 and P1 structure with the one described for T20, (T20+N46) [36], CD spectra of P1 or P5+LZ have been obtained. For both peptides the molar ellipticity [θ] at 222 nm appeared to increase, from -18.05 to -21.32 for the LZ+P1, or from -19.45 to -23.12 for LZ+P5, indicating a slight increase in the α-helical content (Table 1). Upon mixing peptides N36 with P1 or P5, the molar ellipticity at 222 nm was just the sum of the spectra of N36 and P1 or P5. This is in agreement with results obtained when T20 was mixed with NRH peptides [36].

**Table 1:** The α-helical content of P1 & P5 alone or in the presence of N36 & LZ

	[θ]_222_ (10^-3^deg cm^2^dmol^-1^)		[θ]_222_ (10^-3^deg cm^2^dmol^-1^)
N36	-6	LZ	-4
P1	-11	P5	-15
P1+N36	-17	P1+LZ	-21
P5+N36	-21	P5+LZ	-23
^a^T20	-2	^b^T20+N46	-9

**Note**: Peptides were all chemically synthesized; ^a^: from[20]; ^b^: from [36]

### P5, comprising P1 and the gp41 calcium-binding site, inhibits HIV-1 env mediated-cell fusion and infection

Cell fusion experiments are convenient assays for analysis of the mechanism by which peptides inhibit virus entry into host cells. Hela-CD4-LTR-LacZ (P4.2) cells stably express the human CD4 molecule, CXCR4 and long terminal repeat (LTR) driven lacZ gene. Hela-env-Lai cells stably express HIV-Lai envelope glycoproteins gp120 and gp41, and the HIV-1 transactivator Tat. Fusion of Hela-Env-Lai cells and P4.2 cells results in transferring Tat to P4.2 cell cytosol. Tat in turn, transactivates the LTR and initiates transcription of the lacZ gene. The extent of fusion is directly related to β-galactosidase activity in cell lysates [31].

**Figure 3:**
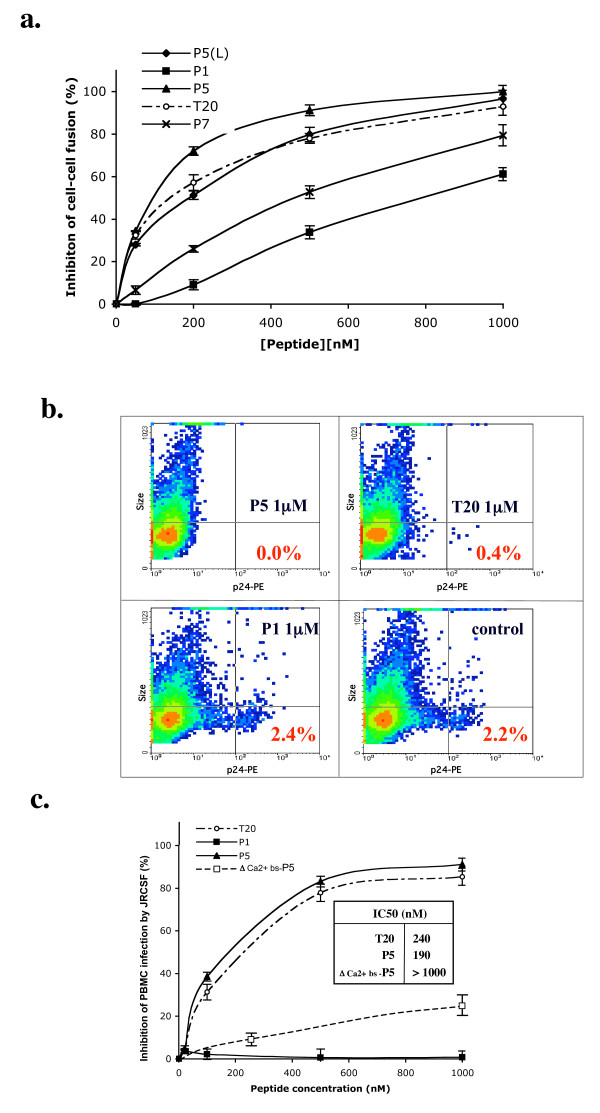
**a. HIV-1 *env *mediated cell-cell fusion inhibition assay**. Cells expressing the HIV-1 envelope were incubated with target cells expressing HIV receptors in the presence of recombinant peptides P5 (black triangle), P1 (black square), P5L (black diamond) and P7 (x), and compared with peptide T20 (open circle). After 6 h of co-culture, fusion was quantified by measuring the beta–galactosidase activity after lysis of the cells. The values were expressed as the average (± S.D.) of three wells from one representative experiment of three. b. Inhibition of human PBMC infection by HIV-Lai in the presence of P1, P5 and T20. Inhibition of PBMC infection by HIV-Lai was evaluated by intracellular staining of p24 Ag. At 1 μM, peptides P5 and T20 exhibited potent inhibition of PBMC infection by X4 virus. In contrast, P1, a shorter peptide devoid of the gp41calcium-binding site, did not inhibit PBMC infection at the same concentration. c. Inhibition of human PBMC infection by HIV-_JRCSF _in the presence of P1, P5 and T20. Percent of inhibition of PBMC infection by HIV-_JRCSF _evaluated as in Figure 3b by intracellular staining of p24 Ag. Inset: IC50 are given in nM.

Different concentrations (≤ 1 μM) of peptides were incubated with HeLa-env-Lai cells in culture medium (DMEM) that contained calcium and magnesium, prior to addition of the P4.2 cells. As shown in Figure 3a and Table 2, P5, P5L and T20 substantially inhibited cell fusion, in contrast to P1, P7 or the peptide LZ comprising the gp41 leucine zipper, which has been reported to be devoid of antiviral activity [17]. Moreover, the P5 peptide encompassing the gp41 lectin- and calcium-binding sites, exhibited the highest antiviral activity of all peptides tested in the calcium-containing medium (Table 2).

**Table 2:** Inhibition of HIV-1 mediated cell-cell fusion by gp41 MPR derived peptides

	IC50_g_ (nM, Mean ± S.D.)		IC50 (nM, Mean ± S.D.)
N36	>1000	LZ	> 1000
P1	913 ± 13.6	P1 + LZ	923.4 ± 11.7
T20	78 ± 5.6	T20 + LZ	579 ± 3.5
P5	60 ± 2.	P5 + LZ	61 ± 1.5
P5L	90 ± 1.7	P7	560 ± 10.7

**Note**: Peptides P5L and P7 are recombinants the other chemically synthesized

Peptides LZ and N36 at 1 μM exhibit negligible inhibition of cell-cell fusion. The presence of LZ did not substantially modify the efficiency with which P5 inhibited cell-cell fusion, in contrast with T20 (Table 2) and in agreement with the data on the structure of the peptides (Table 1). The antiviral properties of peptides P5, P1 and T20 were tested against infection of PBMC by X4- (HIV-1_Lai_) and R5-tropic (HIV-1_JRCSF_) strains of HIV-1. As shown in Figure 3b, and 3c, at 1 μM the P5 and T20 peptides had an inhibitory activity greater than 90% for both X4 and R5 virus infection. IC50 of infection of PBMC by and R5-tropic (HIV-1_JRCSF_) strains was 20% lower for P5 as compared to T20. Strikingly, P5 mutated in the calcium-binding site (Δ Ca2+ bs-P5) had no antiviral activity, suggesting that the calcium-binding site of P5, absent from T20 is determinant in P5 antiviral activity. In contrast, P1 did not inhibit HIV-1 infection of PBMC under the same conditions.

The mechanisms underlying the anti-HIV-1 activity of T20 remain unclear. It has been proposed that T20 directly interacts with gp41 and gp120, thereby inhibiting viral and cellular membrane fusion [36,37]. Moreover, the tryptophan-rich region (a.a.666–673) in T20 could interact with the membrane surface, thereby blocking membrane fusion at a post-lipid mixing stage [36,38]. Although T20 is successfully used in the clinical setting, drug resistant mutants have been reported [19,29], and in particular in the 3 amino acid GIV motif, located in the NHR of gp41, an important binding site for T20.

Peptides derived from the CHR region of the gp41 ectodomain possess strong anti-HIV activity by virtue of interacting with the coiled-coil motif of gp41 [15,39]. Peptide P5 derived from this domain comprises three additional non-polar residues, Trp628, Trp631 and Ile635, as compared with T20 or P1, which could increase the binding affinity of the peptide for a hydrophobic cavity in the coiled-coil region. Additionally, the calcium binding site present in P5 but not in T20 and P1, most likely allow the peptide to adopt an inhibitory conformation in physiological conditions where calcium concentration reaches the micromolar range [20]. Moreover, C-peptides containing the hydrophobic cavity-binding region (a.a.628–635) are much less sensitive to the emergence of resistant virus than T20, which lacks this region [29].

**Figure 4:**
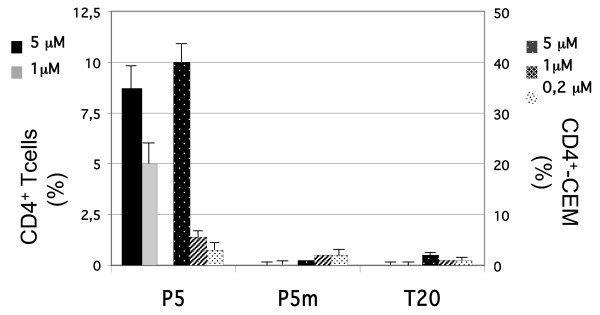
**Binding of Peptides to CD4^+^-target cells**. P5, P5 mutated in the calcium-binding site (P5m), and T20 at indicated concentrations were allowed to interact the CD4^+^-target cells: primary human CD4^+^ T cells or the lymphocytic CD4^+^- CEM cell line. Bound peptide were detected using the IgG 2F5 and analyzed by flow cytometry. Results are shown in a two-scale bar graph (human CD4^+^ T cells (left scale), CD4^+^- CEM cell line (right scale)) as peptide bound cells % the gated population, to which non specific binding has been subtracted.

We next evaluated the respective ability of P5 and T20 to bind directly to the CD4^+^-target cell. As shown on figure 4, P5 bound specifically in a concentration-dependent manner in the micro-molar range to either a CD4^+^ lymphocyte cell line, CEM as well as to primary human CD4^+^ T cells. In contrast, T20 was unable to bind these target cells. These results indicated that T20 and P5 interact differently with the target cell, inferring that each peptide exerts its antiviral activity using a different mechanism. Furthermore, P5 mutated in the calcium-binding site (P5m) was also unable to interact with CD4^+ ^-target cells, indicating that P5 binding to target cells was calcium dependent.

Altogether these data confirm that T20 and P5 exhibit antiviral activities by different mechanism, and that P5 specific activity is calcium dependent.

### P5 inhibits replication of T20-resistant HIV-1 strains

**Figure 5:**
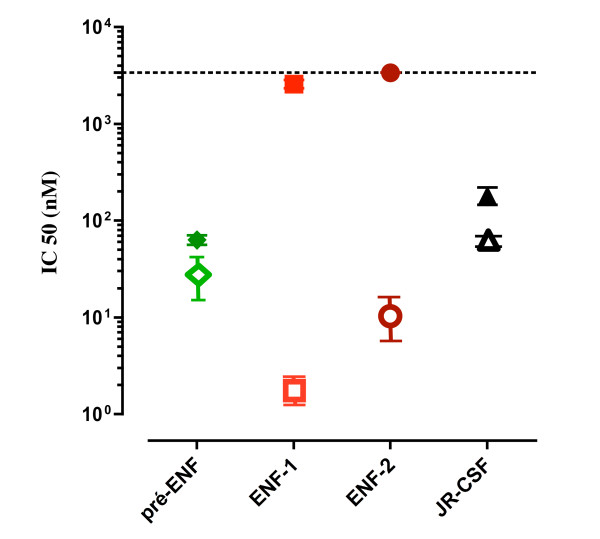
**Inhibition of replication of T20-resistant HIV-1 strains**. Inhibition of the replication of two T20-sensitive (pre-ENF, JRCSF) and two T20-resistant strains (ENF-1 and ENF-2) by P5 was studied in comparison with T20. P5 (open symbols) showed strong inhibitory activities against T20-resistant strains as compared with T20 (closed symbols). Sensitivity to P5 and T20 was measured in a single-cycle assay. Each point represents the mean IC_50 _value of at least three independent experiments (bars represent standard errors).

Since there were significant differences in the anti-HIV activities of P5 and T20, we sought to determine whether P5 is able to inhibit replication of HIV-1 strains resistant to T20. Two T20-sensitive (pre-ENF and JRCSF) and two T20-resistant strains (ENF-1 and ENF-2), with well-defined genetic mutations conferring the resistance, were used in our experiments [30]. The envelope of two T20-resistant strains, ENF-1 and ENF-2, were cloned from virus carried by the patient undergoing T20 treatment that eventually failed. They were compared with the envelope of virus recovered from the patient prior to T20 treatment (pre-ENF), and with that of JRCSF. We found that both P5 and T20 were active against T20-sensitive strains, but that P5 was more potent than T20, consistently with our observations (section 3.2) regarding the inhibition of infection of PBMCs by JR-CSF or Lai virus. More importantly, P5 showed strong inhibitory activities against all T20-resistant strains with IC50 ranging from 2 to 9 nM, while T20 failed to inhibit even at concentrations as much as 250- to 2000-fold higher (Figure 5). These results suggest that P5 could be used as an alternative fusion inhibitor for treatment of patients with HIV-1/AIDS, in particular those infected by T20-resistant variants.

Several non-exclusive factors may explain the strong anti-HIV-1 activity of P5 reported here. Amino acids 628 to 663 of peptide P5, containing the calcium-binding site, are essential for binding to the NHR region. It may overcome the interaction with the three non-polar residues GIV that uses T20 for direct interaction with viral gp41 NHR, thereby disrupting the six-helix bundle formation. Furthermore, it would explain how P5 exhibited an antiviral activity against the T20-resistant viruses ENF-1 and -2 that lack this GIV motif. P5 also comprises a lipid-binding domain (666–673) essential for maintaining the anti-HIV-1 activity of T20 [17,36]. In this regard P5 would be similar to other peptides from the C-terminal heptad repeat that are thought to act as strong anti-HIV-1 activity by binding directly to the virus, thereby preventing hairpin formation and subsequent inhibition of fusion between viral and cellular membranes. Moreover, since P5 harbors a calcium-binding site [20], in the presence of extracellular calcium P5 might adopt a different conformation due to the saturation of the calcium binding site. Indeed, the P5 peptide, shown to be able to bind directly to the target cell, whereas the mutated P5 did not bind, could compete with the incoming viral particle and thereby inhibit fusion. Accordingly, as shown on Fig. 3c, a P5 peptide with mutation in the amino acids forming the calcium-binding site [21] exhibited minimal inhibition of CD4^+^ T cell infection by the R5-tropic JR-CSF virus as compared with wild type P5 (inhibition at a peptide concentration of 250 nM was negligible for mutated P5 (8%) as compared to 65% for wild type P5). This suggests that the superior antiviral activity of P5, as compared with T20, could be attributable to the calcium-binding site conformation along with the tryptophan-rich hydrophobic region immediately adjacent to the viral membrane. These structural elements could confer an efficient antiviral activity, and raise the possibility that calcium plays a role in peptide conformation and its direct interaction with the membrane, either of the virus or the target cell.

In contrast, peptide P1 contains only a partial CHR region without the calcium-binding site and the hydrophobic cavity-binding domain and does not exhibit any anti-HIV-1 activity. The low antiviral activities of P7 peptide indicated that the C-terminal cytoplasmic tail in P7 did not contribute to the antiviral activities. Previous research suggested that C-peptides that had a low tendency to adopt a helical conformation failed to bind to the coiled-coil motif and in turn did not prevent gp41-mediated cell fusion [40,41].

Accordingly, the CD analysis indicated that peptides P1 and P7 with low anti-HIV-1 activity exhibited lower helical content compared with peptides P5 and P5L with high antiviral activity. The high helical content and the three non-polar residues facing the hydrophobic cavity, together with the structure of the calcium-binding site, are therefore likely to be responsible for the high anti-HIV activity of peptides P5.

In the present study, we found that the antiviral activities of recombinant peptides P1, P5 and P5L were similar to those of their chemically synthesized counterparts (not shown), that pave the way for an easier development of the use of long peptides, usually difficult and costly to produce, as antivirals. Thus recombinant peptides hold promise for HIV-1 salvage treatment in resource-poor settings. Moreover, the expression of recombinant peptides P1, P5 and P5L should enable structural analysis by NMR under different conditions. In particular, study by NMR of isotopically labeled recombinant P1 and P5 in the presence of liposomes of different lipid composition [42] and of neutralizing antibodies should provide structural information of use in HIV-1 vaccine design. However, generation of escape mutants have restricted the use of T20; and that could apply to other currently approved anti HIV medications. Furthermore, the use of peptides as therapeutics could also be restricted by immunogenicity and half-life in circulation. Hence all these parameters need to be met before a peptide can be a therapeutic.

## Competing interests

The authors declare that they have no competing interests

## Authors' contributions

HY participate to the conception of the study, carried out the molecular biology, biophysical studies and neutralization assays, and drafted the manuscript. DT participated to the neutralization and binding assays. AA participated in the design of the study and writing of the manuscript. BL and FC performed the neutralization assays involving ENF-resistant viruses. MB conceived of the study, and participated in its design and coordination and writing of the manuscript. All authors read and approved the final manuscript.

## Supplementary Material

Additional File 1 **Expression and purification of recombinant peptides**. The data provided describe in details the methodology for expression and purification of the various recombinant fusion peptides used in the study. Click here for file
