# Supplementary material for: Peptide P5 (residues 628–683), comprising the entire membrane proximal region of HIV-1 gp41 and its calcium-binding site, is a potent inhibitor of HIV-1 infection
Source: Retrovirology. 2008 Oct 16;5:93. doi: 10.1186/1742-4690-5-93 (PMC2585100; doi:10.1186/1742-4690-5-93)
Supplement: Additional File 1 — Expression and purification of recombinant peptides. The data provided describe in details the methodology for expression and purification of the various recombinant fusion peptides used in the study. [file 1742-4690-5-93-S1.pdf]

Supplementary data

Expression and purification of recombinant fusion peptides

A. Recombinant fusion peptides were analyzed by10-20% gradient SDS-PAGE gel with Coomassie blue staining. Lane 1, 2, 3, 4: purified fusion peptides P7, P1, P5, P5L respectively; lane 5: *E. coli Rosetta*™-pGEV, lane 6, 7, 8, 10: *E. coli Rosetta*™-pGEV-P7, -pGEV-P5, pGEV-P1, pGEV-P5L after IPTG induction; The size of protein molecular weight marker (MultiMark, invitrogen) are indicated. **B.** Expression of recombinant fusion peptides was analyzed by western blot staining. Lane 1, 3,5, 7: Induction of *E. coli* Rosetta™ cells carrying constructions P1, P5, P5L and P7 for 3h, respectively; Lane 2, 4, 6, 8 induction for overnight. **C.** Growth of *E. coli* Rosetta™ cells carrying different constructions. The toxicity of the expression proteins to *E. coli* Rosetta™ was evaluated by measuring the optical densities at 600nm of induced culture cells at the indicated times (hours). **D.** Reverse phase HPLC for P1, P5 was analyzed by using a C18 column. The peak that corresponds to peptides P1 (peak a) and peptides P5 (peak b) is eluted about 40min; the run was monitored at 280nm.

Comment

Since the peptides P5 is highly hydrophobic, production of P5 in baculovirus appeared difficult to obtain (Morgane Bomsel, unpublished data). We also found that inserting the P5 gene fragment into the pET-16b expression vector could not result in P5 production in *E.Coli* Rosetta™ probably due to the toxicity of P5 for both prokaryotic and euokaryotic cells.

Here, the successful production of the hydrophobic gp41-MPR-derived peptides in *E.Coli* was shown using the GB1 (B1 immunoglobulin-binding domain of streptococcal protein G) fusion protein system.

Construct and expression the hydrophobic peptides derived from MPR of gp41

To obtain the hydrophobic peptides, many strategies rely on the use of fusion protein tags to improve expression as well as to facilitate purification. It also helps in peptide stability

and solubility following expression. Here, the GB1 fusion protein system was chosen as expression system for improving expression [1, 2]. The small size of GB1 allows to perform additional spectroscopic analysis [3]. Moreover, the GB1 fusion protein expression vector (pGEV vector) contains a thrombin-cleavable linker between the GB1 domain and the desired protein. It allows purification by affinity chromatography using IgG-sepharose and desired protein can be cleaved enzymatically from the fusion protein. The possibility of expressing milligram quantities of proteins will be useful for further structure determination and anti-HIV-1 activities study.

The gene fragments encoding P5, P1, P5L and P7 carrying the transmembrane region (TM) were respectively inserted into pGEV vector that contains a GB1 domain, a thrombin cleavable linker. The sequence of recombinant plasmids pGEV-P1, pGEV-P5, pGEV-P5L and pGEV-P7 were confirmed by restriction enzyme analysis and by DNA sequencing (data not shown).

These recombinant plasmids were transformed into *E. coli* Rosetta™ and induced the production for 3h, overnight, respectively. The Rosetta™ cell line helps to alleviate codon bias and enhance expression of heterologous proteins. The 10-20% gradient SDS-PAGE gel analysis suggests that fusion peptides P5, P1 and P5L have higher expression as compared to P7 (Supplementary data **A**). Western blot analysis indicated that recombinant fusion peptides P5, P1, P5L, P7 could specifically react with IgG 2F5. Moreover, 3h IPTG induced bacterial cultures resulted in higher production as compared to overnight culture (Supplementary data B). However, formation of recombinant fusion peptides P5 and P5L oligomers was detected in overnight cultures due to their hydrophobic characteristic or the aggregation of the encoded protein as inclusion bodies.

Measurement of the optical densities at 600nm of induced *E. coli* Rosetta™ cells at 1h, 2h, 3h suggested that the expression P7 is toxic to *E. coli* Rosetta™ (Supplementary data C).

The P7 contains P5L as well as the long transmembrane region (a.a.683-746). As compared to P5L, the low production of P7 may result from the transmembrane region (TM) toxicity.

After lysis of induced bacterial cultures, recombinant peptides were detected both insoluble and soluble fractions. Purification of the peptide from the soluble fraction by affinity chromatography could yield approximately 1-3mg of peptides per 1 liter of bacterial cultures. The purified peptides were next analyzed by SDS-PAGE followed by Coomasie Brilliant Blue staining, and was found homogeneous at expect molecular mass (Supplementary data A).

The fusion proteins were each bound to a sepharose IgG column in TST buffer (pH 7.6; 50mM Tris , 150mM NaCl , 0.05% Tween-20), followed by 10 washes in TST, and a final wash in PBS. The peptides were then released from the column-bound recombinant fusion-proteins by enzymatic cleavage with thrombin (10 IU/mg of protein, 500 microliter) in an incubation of 16hr at 22 °C. To eliminate thrombin from the eluate containing the cleaved peptides, the eluate was directly loaded onto a Benzamidine FF column, equilibrated in 20mM Na phosphate, 0.15M NaCl, pH 7.5, which binds thrombin. The subsequent eluate was collected in 0.5-1ml fractions that contained the pure protein of interest. After on-column cleavage and purification, the peptides P1 and P5 were analyzed by reverse phase RP HPLC. The peptides P1 and P5 with a high purity were eluted at about 40min corresponding to peak (a) and peak (b) respectively (Supplementary data D). The collected fraction of the peak (a) and (b) was analyzed by MALDI-TOF mass spectrometry and provided the molecular mass of 4899.44Da for P1 and 7503.3Da for P5, close to the predicted mass of 4901.4Da for P1 and 7504.4Da for P5.

1. Gronenborn, A.M., D.R. Filpula, N.Z. Essig, A. Achari, M. Whitlow, P.T. Wingfield, and G.M. Clore, *A novel, highly stable fold of the immunoglobulin binding domain of streptococcal protein G.* Science, 1991. **253**(5020): p. 657-61.

2. Lindhout, D.A., A. Thiessen, D. Schieve, and B.D. Sykes, *High-yield expression of isotopically labeled peptides for use in NMR studies.* Protein Sci, 2003. **12**(8): p. 1786-91.
3. Huth, J.R., C.A. Bewley, B.M. Jackson, A.G. Hinnebusch, G.M. Clore, and A.M. Gronenborn, *Design of an expression system for detecting folded protein domains and mapping macromolecular interactions by NMR.* Protein Sci, 1997. **6**(11): p. 2359-64.

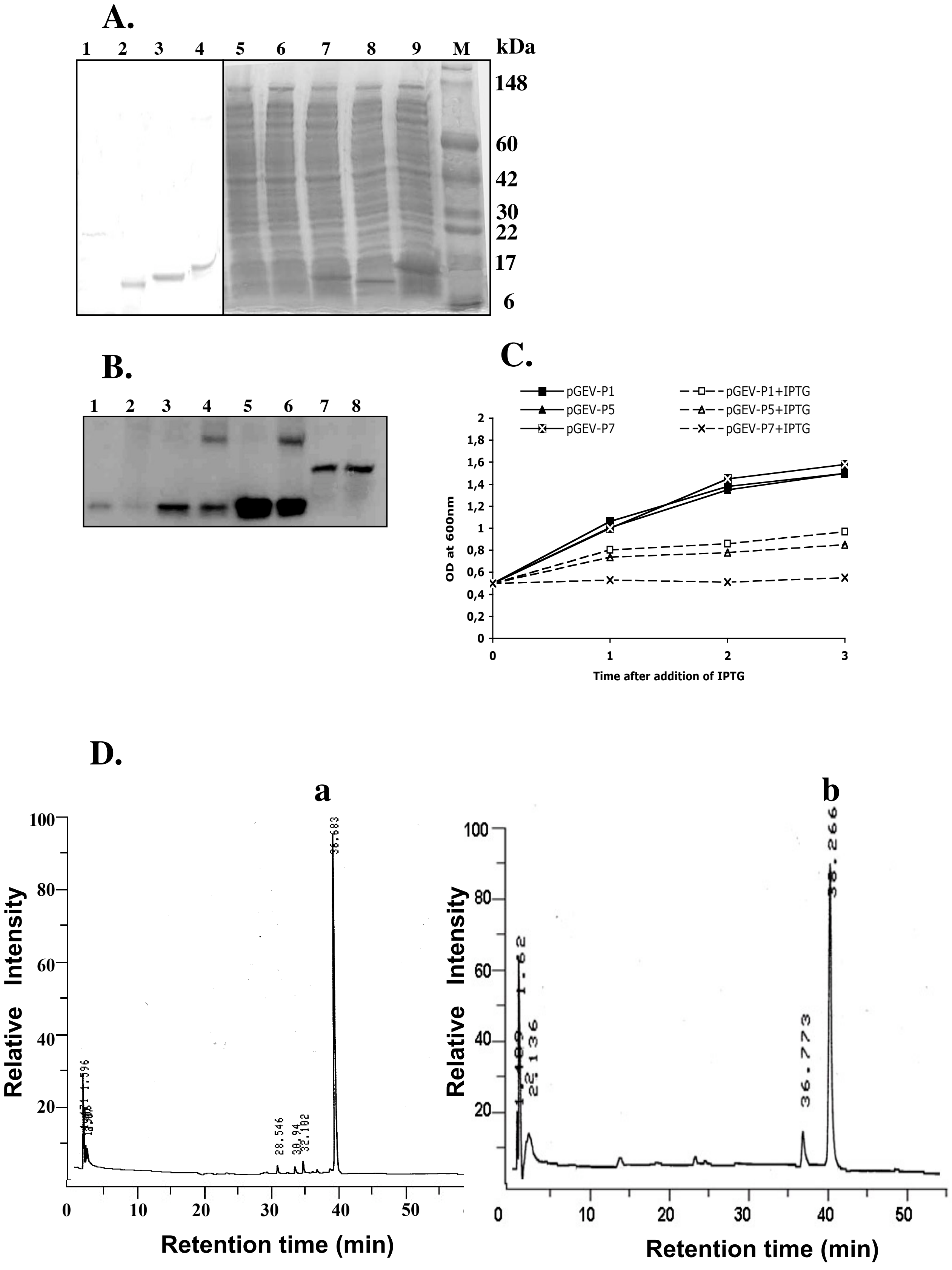

Supplementary data
